# 미인증 종합병원간호사의 환자안전관리 중요성 인식, 환자안전역량이 환자안전간호활동에 미치는 영향

**DOI:** 10.7586/jkbns.24.002

**Published:** 2024-02-26

**Authors:** Ji-Yeong Park, Hanna Choi

**Affiliations:** 1 Patient Safety Management Office, Bitgaram General Hospital, Naju, Korea; 1 빛가람종합병원 환자안전관리실; 2 Department of Nursing Science, Nambu University, Gwangju, Korea; 2 남부대학교 간호학과

**Keywords:** Patient safety, Risk management, Awareness, General hospitals, 환자안전, 위험관리, 인식, 종합병원

## Abstract

**Purpose:**

This descriptive research study attempted to determine how general hospital nurses’ awareness of the importance of patient safety management and patient safety competency affected patient safety management activities.

**Methods:**

From September 13 to 26, 2022, a survey was administered to 230 ward nurses who provided direct care to patients at five non-accredited general hospitals being evaluated for accreditation located in metropolitan cities. The collected data were analyzed with descriptive statistics, the t-test, one-way analysis, the Scheffé test, Pearson correlation coefficients, and hierarchical regression using SPSS for Windows version 26.0.

**Results:**

In total, 221 (96.1%) respondents were female. The average age was 32.2 years, and the average clinical experience was 3.5 years; 196 participants (85.2%) were general nurses. Patient safety competency (β = .44, *p* < .001), awareness of the importance of safety management (β = .31, *p* < .001), and medication error experience (β = -.15, *p* = .002) all had statistically significant associations with patient safety management activities. The explanatory power of these variables for patient safety management activities was 50.7%.

**Conclusion:**

This study confirmed that patient safety competency, awareness of the importance of patient safety management, and experience with medication errors significantly influenced patient safety management activities.

## 서론

### 1. 연구의 필요성

최근 의료 환경은 급격한 고령화에 따라 삶의 질에 대한 관심이 높아지면서 의료기관 질 향상에 대한 국민적 요구가 증대되었다[1]. 이에 정부는 의료서비스 수준의 평가를 통해 환자안전과 의료서비스의 질을 향상시키고자 인증제도를 도입하여 의료기관을 대상으로 인증평가를 실시하고 있다[2]. 의료기관평가인증원에서 발표한 자료에 따르면 327개의 종합병원 중 의료기관 인증을 획득한 곳은 203곳인 62%에 불과하였다[2,3]. 보건의료기관의 종별 환자안전사고 발생 현황은 종합병원의 환자안전사고 보고 건수가 2021년 5,163건으로 전년 대비 203건 증가하였으며, 이는 보건의료기관 중 가장 높은 보고 건수를 기록하였다[3]. 특히 의료기관 인증제를 받지 않은 종합병원은 환자안전관리 중요성에 대한 인식이 낮고, 교육이 제대로 이루어지지 않은 것으로 조사되어 미인증 종합병원에 더 큰 관심이 요구되는 실정이다[4]. 반면 상급종합병원에서는 의료기관평가가 의무 평가방식으로 의료서비스에 대한 수준 향상, 의료기관의 관심, 임상 질 지표 도입 등의 성과를 이루고 있다. 상급종합병원과 종합병원에서 인증평가를 경험한 간호사군에서 환자안전간호활동이 증가한 것이 대표적인 사례 중의 하나이다[5].

간호사의 환자안전간호활동은 간호사가 환자에게 사고 혹은 위해 사건을 예방하는 것으로 환자안전을 증진시키기 위하여 실시하는 관리활동이다. 여기에는 감염, 낙상, 욕창 예방, 환자확인, 구두 및 전화 처방, 투약 간호, 수술 및 시술간호, 안전한 환경 관리, 수혈간호, 응급상황대처 등을 포함한다[6]. 특히 환자안전과 관련된 모든 행위를 주체하는 간호사는 의료서비스 질 측면에서 차지하는 비중이 크므로 그 중요성이 더욱 강조된다[7]. 환자안전간호활동에 간호사의 환자안전관리 중요성 인식이 긍정적인 영향을 미치며[8], 환자안전 관련 문제의 확인 및 해결과정에서도 중요한 요인으로 알려졌다. 이는 간호사가 먼저 환자안전관리의 중요성을 인식하고, 직접적으로 관리함으로써 간호업무 질을 향상시킬 수 있기 때문이다. 따라서 효율적인 환자안전간호활동을 위해 환자안전관리 중요성 인식 강화가 우선으로 요구된다[9].

환자안전간호활동과 관련하여 간호근무환경, 환자안전관리 중요성 인식, 환자안전문화, 조직의사소통, 조직몰입, 자기효능감 등 영향요인에 대한 연구가 활발하게 이루어지고 있다[6,9]. 간호사의 직무별 환자안전간호활동에 관한 선행연구를 살펴보면, 응급실 간호사와 중소병원 간호사의 환자안전간호활동을 증진시키기 위해 환자안전시스템 개선방안 필요성이 제기되었다[10,11]. 간호·간병통합서비스 간호사는 환자안전 지식을 높이고, 긍정적 태도를 함양할 때 수행률이 높게 나타났다[12]. 요양병원 간호사의 환자안전간호활동에 영향을 주는 요인은 안전통제감, 사건 보고에 대한 태도 중 보고의도, 조직에서의 의사소통 중 비공식적인 의사소통으로 확인되었다[13]. 그러나 종합병원간호사를 대상으로 환자안전관리 중요성 인식과 환자안전역량이 환자안전간호활동에 미치는 영향을 확인한 연구는 미비한 실정이었다. 더 나아가 의료기관 인증을 획득하지 않아 환자안전 관리 여부가 확인되지 않은 미인증 종합병원에 근무하는 간호사를 대상으로 환자안전간호활동에 대한 관련성을 확인한 연구도 찾아보기 힘들다.

환자안전사고를 예방하고 안전한 의료를 제공하기 위해 의료인이 갖추어야 할 지식, 기술, 태도를 의미하는 환자안전역량은 불필요한 위해의 위험으로부터 환자를 보호하기 위해 간호 인력이 갖추어야 할 필수 요소로 꼽힌다[14]. 따라서 환자안전을 선택이 아닌 필수로 여기고 의료 오류로 인한 안전에 대해 경각심을 높이고 위험도는 낮추어 환자중심의 안전한 서비스를 제공할 수 있도록 돕는 환자안전관리 중요성 인식과 환자안전역량이 환자안전간호활동 수행에 중요하다[15].

이에 본 연구는 의료기관 인증평가에 참여하지 않은 종합병원에 근무하고 있는 간호사를 대상으로 환자안전관리 중요성 인식과 환자안전역량이 환자안전간호활동에 미치는 영향을 규명하여, 종합병원간호사의 환자안전간호 활동을 향상시킬 수 있는 기초자료를 제시하여 임상간호학에서 요구되는 안전간호와 관련된 기초간호학적 지식을 수립하기 위하여 시도되었다.

### 2. 연구 목적

본 연구는 종합병원간호사의 환자안전관리 중요성 인식과 환자안전역량이 환자안전간호활동에 미치는 영향을 파악하기 위함이며, 구체적인 목적은 다음과 같다.

1) 대상자의 일반적 특성을 파악한다.

2) 대상자의 환자안전관리 중요성 인식, 환자안전역량 및 환자안전간호활동 정도를 파악한다.

3) 대상자의 일반적 특성에 따른 환자안전간호활동의 차이를 파악한다.

4) 대상자의 환자안전관리 중요성 인식, 환자안전역량 및 환자안전간호활동 간의 상관관계를 파악한다.

5) 대상자의 환자안전간호활동에 영향을 미치는 요인을 파악한다.

## 연구 방법

### 1. 연구 설계

본 연구는 종합병원 간호사를 대상으로 환자안전관리 중요성 인식, 환자안전역량이 환자안전간호활동에 미치는 영향을 파악하고자 시도된 서술적 조사연구이다.

### 2. 연구대상 및 자료수집

본 연구는 전라남도 나주시와 광주광역시 소재 100∼500병상 규모의 5개 병원인증평가 미인증 종합병원에 임상경력 3개월을 초과하여 근무하는 간호사들을 대상으로 하였으며, 특수부서 및 외래부서는 병동과 실무영역이 달라 부서에 따른 환자안전활동에 차이가 있다는 선행연구를 참고하여 제외하였다[16]. 표본의 크기는 G*Power 3.1.3 프로그램을 이용하여 단계적 다중회귀분석에서 중간효과 크기 .15, 검정력 .95, 유의수준 .05 기준으로 하였을 때, 요구되는 최소 표본의 수는 204명이었다. 이에 탈락률 20%를 고려하여 245명을 목표로 하였으며, 총 245부 설문지를 배포하여 235부(96%)가 회수되었다. 누락된 항목이 2개 이상인 경우와 일률적으로 같은 번호만 기입하는 등 응답이 불성실한 5부를 제외한 230부를 최종 분석하였다.

### 3. 연구 도구

#### 1) 환자안전관리 중요성 인식

환자안전관리 중요성 인식의 측정 도구는 Park 과 Kim [17]이 개발한 것을 사용하였다. 이 도구는 총 21문항 4개 하위영역으로 ‘환자안전관리에 대한 인지’가 4문항, ‘환자안전관리에 대한 관심’이 7문항, ‘환자안전관리에 대한 의지’가 5문항, ‘환자안전관리에 대한 자신감’이 5문항 등으로 구성되었다. 개발 당시 측정도구의 신뢰도 Cronbach's α는 .86이었고, 본 연구에서의 Cronbach's α는 .93이었다.

#### 2) 환자안전역량

환자안전역량은 Jang [14]이 간호대학생을 대상으로 개발한 도구를 간호사를 대상으로 적용한 Lee [18]의 도구를 타당도와 신뢰도를 검증한 도구를 사용하였다. 이 도구는 총 41문항으로 지식, 기술, 태도 3개 영역으로 구성되었다. 환자안전태도 2문항은 역으로 환산하고, 그 외에 각 문항은 Likert 5점 척도로 점수가 높을수록 환자안전에 대한 역량이 높음을 의미한다. 도구의 신뢰도는 도구개발 당시 Cronbach's α는 .90이었고, 본 연구에서의 Cronbach's α는 .95이었다.

#### 3) 환자안전관리활동

환자안전간호활동은 Han 과 Jung [5]이 의료기관평가인증원(인증평가기준집 ver 2.0) [19]의 평가 기준을 토대로 개발한 도구를 사용하였다. 이 도구는 총 32문항 8개 하위영역으로 ‘정확한 환자확인’ 4문항, ‘의사소통’ 4문항, ‘수술/시술 전 환자안전’ 3문항, ‘낙상예방활동’ 6문항, ‘손 위생 및 감염관리’ 5문항, ‘화재안전 및 응급상황관리’ 2문항, ‘투약’ 6문항, ‘시설 및 의료기기관리’ 2문항 등으로 구성되었다. 각 문항은 Likert 5점 척도로 측정하며, 점수가 높을수록 환자안전간호활동에 대한 수행 정도가 높음을 의미한다. 개발 당시 측정도구의 신뢰도 Cronbach's α는 .95이었고, 본 연구에서의 Cronbach's α는 .91이었다.

### 4. 자료분석 방법

수집된 자료는 SPSS/WIN 26.0 프로그램(IBM Corp., Armonk, NY, USA)을 이용하여 통계 분석하였다.

• 대상자의 일반적 특성은 빈도와 백분율, 평균과 표준편차로 분석하였다.

• 대상자의 환자안전관리 중요성 인식, 환자안전역량, 환자안전간호활동의 정도는 평균과 표준편차로 분석하였다.

• 대상자의 일반적 특성에 따른 환자안전관리 중요성 인식, 환자안전역량 및 환자안전간호활동의 차이는 t-test와 one-way ANOVA를 이용하였고, 사후검정은 Scheffé test로 분석하였다.

• 환자안전관리 중요성 인식, 환자안전역량 및 환자안전간호활동 간의 상관관계는 Pearson correlation coefficient로 이용하여 분석하였다.

• 환자안전간호활동에 대한 영향요인은 hierarchical regression으로 분석하였다.

### 5. 윤리적 고려

본 연구는 2022년 9월 광주광역시 남부대학교의 IRB 심의를 통과한 후 (IRB No. 1041478-2022-HR-016) 시행되었다. 윤리적 고려를 위하여 본 연구자는 연구대상자용 설명문 및 동의서를 활용하여 대상자에게 연구 목적과 연구 내용 및 연구 참여로 인한 불편감, 신분의 비밀보장과 사생활 보호, 연구 과정 중에도 원하지 않는 경우 바로 중단할 수 있음을 설명하고 설문지 작성 전 동의서에 연구대상자가 직접 서명을 하도록 하였다. 수집된 자료는 잠금장치가 있는 곳에 보관하였으며, 대상자 고유번호를 부여하여 코딩된 엑셀파일은 비밀번호가 설정된 컴퓨터에서 보관하여 개인정보를 보호하였다.

## 연구 결과

### 1. 일반적 특성

연구대상자는 총 230명으로 성별은 여성이 221명(96.1%)으로 대부분을 차지하였다. 평균연령은 32.2세이었고, 결혼상태는 미혼이 155명(67.4%), 최종학력은 대학교 졸업이 151명(65.7%), 총 임상경력은 10년 이상이 65명(28.2%)으로 가장 많았다. 직위는 일반간호사가 196명(85.2%), 근무형태는 3교대 근무자가 186명(80.9%)으로 나타났다. 대상자 중 환자안전간호에 대한 교육경험이 ‘있다’가 205명(89.1%), 지난 12개월 동안 환자안전사고 보고 건수는 1∼2회가 125명(54.3%), 지난 1년간 직·간접 환자낙상경험은 ‘없다’가 146명(63.5%)이었다. 투약오류경험은 ‘없다’가 155명(67.4%), 검사오류경험은 ‘없다’가 210명(91.3%), 수술/처치/시술오류경험은 ‘없다’가 216명(93.9%), 환자안전관련 업무는 ‘없다’가 219명(95.2%)이었다(Table 1).

### 2. 환자안전관리 중요성 인식, 환자안전역량 및 환자안전간호활동의 정도

대상자의 환자안전관리 중요성 인식 정도는 3.92 ± 0.47점이었다. 환자안전역량 정도는 3.92 ±0.42점으로 나타났고, 환자안전간호활동 정도는 4.30 ± 0.43점으로 나타났으며, 세 변수의 하위영역별 점수는 Table 2와 같다.

### 3. 일반적 특성에 따른 환자안전관리 중요성 인식, 환자안전역량 및 환자안전간호활동의 차이

환자안전관리 중요성 인식은 연령(F = 3.22, *p* = .042)에 따라 통계적으로 유의한 차이가 있었다. 유의한 변수에 대해 사후 검정한 결과 40세 이상이 4.07 ± 0.50점으로, 30~39세 3.84 ± 0.46점보다 환자안전관리 중요성 인식이 더 높은 것으로 나타났다. 직위(F = 4.11, *p* = .018)에 따라 통계적으로 유의한 차이가 있었다. 유의한 변수에 대해 사후 검정한 결과 수간호사가 4.20 ± 0.48점으로 책임간호사 3.79 ± 0.45점보다 환자안전관리 중요성 인식이 더 높은 것으로 나타났다. 환자안전역량과 직위(F = 3.64, *p* = .028), 환자안전관련업무(t = 2.57, *p* = .011)와 통계적으로 유의한 차이가 있었다. 수간호사, 환자안전관련업무 경험이 없는 그룹에서 환자안전역량이 높은 것으로 나타났다. 환자안전간호활동은 지난 12개월 동안 안전사고 보고 건수(F = 3.85, *p* = .010), 투약오류경험(t = 3.50, *p* = .001)과 통계적으로 유의한 차이가 있었다(Table 3).

### 4. 환자안전관리 중요성 인식, 환자안전역량 및 환자안전간호활동 간의 상관관계

환자안전관리 중요성 인식은 환자안전역량(r = .62, *p* < .001), 환자안전간호활동(r = .60, *p* < .001)과 유의한 양의 상관관계를 보였다. 환자안전역량은 환자안전간호활동(r = .64, *p* < .001)과 유의한 양의 상관관계를 나타냈다(Table 4).

### 5. 환자안전간호활동 영향요인

본 연구에서 회귀분석을 위한 오차의 자기상관과 다중공선성을 검토하였다. 그 결과 Durbin-Watson을 이용한 오차의 자기상관은 1.94로 검정통계량 2에 가까우므로 자기상관이 없이 독립적인 것으로 확인되었다. 변수들 간의 다중공선성 여부를 검정한 결과, 분산팽창지수(VIF)는 1.089~1.65으로 10.0보다 크지 않았고, 공차한계(Tolerance)는 0.60~0.91로서 0.1 이상의 값을 보여 다중공선성의 문제가 없는 것으로 나타났다. 또한 정규성과 선형관계의 가정을 만족하였고, 특이값을 검토하기 위한 Cook’s 거리를 확인한 결과 개체 중 1.0 이상인 개체는 발견되지 않아 분석을 시행하기 위한 기본 가정은 충족되었다.

환자안전간호활동에 대한 영향 정도를 파악하기 위하여 위계적 다중회귀분석을 실시하였다. 1단계에서는 대상자의 일반적 특성 중 환자안전간호활동에 유의한 차이를 보인 지난 12개월 보고건수, 투약오류경험을 투입하여 분석하였다. 그 결과 투약오류경험(ß = -.20, p = .004) ‘없다’가 환자안전간호활동이 높게 나타났으며, 설명력은 5.2%이었다. 위계적 회귀분석 2단계에서는 1단계에서 투입한 지난 12개월 안전사고 보고 건수, 투약오류경험을 통제한 후 환자안전간호활동과 유의한 상관관계를 나타낸 환자안전관리 중요성 인식, 환자안전역량을 추가로 투입하였다. 그 결과 환자안전간호활동에 가장 높은 영향을 준 변수는 환자안전역량(ß = .44, *p* < .001)였으며, 그 다음은 환자안전관리 중요성 인식(ß = .31, *p* < .001), 투약오류경험(ß = -.15, *p* = .002)의 순으로 나타났다. 모델2의 설명력은 50.7%로 모델1의 설명력보다 45.5% 증가하였다(F = 59.83, *p* < .001) (Table 5).

## 논의

본 연구는 미인증 종합병원간호사의 환자안전관리 중요성 인식, 환자안전역량이 환자안전간호활동에 미치는 영향을 규명하기 위해 실시하였다. 본 연구의 결과, 환자안전관리 중요성 인식에는 연령, 직위가 통계적으로 유의한 차이를 보였다. 연령과 직위가 높을수록 환자안전관리 중요성 인식은 높은 것으로 나타났다. 연령이 많을수록 직위가 높아지고 업무에서 차지하는 역할 비중과 책임감이 커짐에 따라 환자안전 중요성 인식 정도가 높아졌음을 유추할 수 있다[9]. 따라서 환자안전관리 중요성 인식 증진을 위한 교육을 실시할 경우, 연령과 직위를 고려하여 환자안전관리에 대한 이해도를 먼저 확인하고 교육의 빈도와 난이도를 달리할 필요가 있다.

미인증 종합병원간호사가 지각한 환자안전관리 중요성 인식은 다른 종합병원간호사를 대상으로 한 선행연구 결과보다는 높았으나, 상급종합병원 대상 간호사보다는 낮은 결과를 보였다[9]. 이는 본 연구대상자들이 코로나19 팬데믹을 겪으면서 감염병 등 환자안전에 관한 관심과 경각심이 높아져 환자안전관리의 중요성 인식과 환자안전간호활동으로 연결된 것으로 사료된다. 또한 의료기관 인증평가가 필수적인 상급종합병원에서의 체계적인 신규 간호사교육과 의료기관 인증 경험 등이 쌓여 환자안전관리 중요성 인식 정도가 종합병원간호사와 비교하여 높은 수준이라는 선행연구를 반영하였다[4,8]. 이런 의료기관 인증 경험이 상급종합병원에 근무하는 간호사에게 환자안전에 대한 관심 증대와 더불어 환자가 호소하는 문제의 중증도에 따른 높은 환자안전 간호수행 요구도가 학습의 기회를 주었을 것이라 사료된다.

본 연구 결과 환자안전간호활동은 종합병원간호사를 대상으로 한 선행연구와 비슷한 수준으로[6,20] 손 위생 및 감염관리가 가장 높고, 화재안전 및 응급상황관리, 의사소통 순으로 낮아졌다[5,6,16]. 실제 의료기관 평가기준에 화재안전 관리활동 항목(화재예방 및 점검계획, 소방 훈련 및 안전교육, 직원 대응 체계 숙지 여부)에 대한 조사를 포함하고 있으나, 평가에 대한 객관적인 기준이 없어 실태점검이 어려운 실정이다. 또한 인증조사기준집에서 메뉴얼 및 훈련계획 점검 수준이며[21], 화재예방과 같이 대규모 훈련을 실시하기 어렵고, 직접 간호와 연관성이 떨어져 관심의 정도가 낮은 것이 영향을 주었을 것이라고 유추해 볼 수 있다[22]. 따라서 화재훈련 및 응급상황 발생 시 인명 피해가 발생하지 않도록 인명 대피 훈련, 야간시간 피난 훈련 등 피난 안전성 확보를 위한 실질적이고 반복적인 모의 훈련이 강조된다.

의사소통은 Baek [15]의 연구결과와 유사하게 환자안전간호활동의 세부 영역 중 두 번째로 점수가 낮게 조사되었다. 부정확한 의사소통은 간호사들의 조직 의사소통 만족도를 저하시키고, 실수를 유발하여 환자안전에 대한 태도에도 영향을 주어 환자안전을 위협하는 주요한 영향요인이 될 수 있기 때문이다[11]. 따라서 안전한 병원 환경을 만들기 위하여 물리적인 시설지원에서 더 나아가 의료 과오에 대하여 개방적으로 의사소통하고 협력하는 분위기를 만드는 것이 중요할 것이다[23]. 또한 환자 수에 비해 불충분한 인력배치로 인한 간호사의 과다한 업무량이 원활한 의사소통을 막는 것을 방지하고[16], 의사소통 향상을 위해 간호정보기록 시스템의 적극적인 활용과 업무 프로세스 변경 및 개선을 통한 표준화된 의사소통 전략을 실천이 중요하다고 본다.

환자안전간호활동은 지난 12개월 동안 환자안전사고 보고 건수, 투약오류경험 유무가 통계적으로 유의한 차이를 보였다. 지난 12개월 동안 환자안전사고 보고 경험과 투약오류경험이 없을 때 환자안전간호활동이 높은 것으로 나타났다. 선행연구에서는 환자안전사고를 경험한 대상자가 과반수이며, 투약오류경험이 있는 것으로 본 연구결과와 다르게 조사되었다[16]. 환자안전보고학습시스템에 따르면, 환자안전보고체계가 시행된 2016년 7월부터 2021년 12월까지 접수된 총 환자안전사고는 총 52,132건으로 조사되었고 2018년 9,250건, 2019년 11,953건, 2020년 13,919건, 2021년 13,146건으로 전년대비 증감 보고 건수로 773건 감소하였다[3]. 이는 성공적인 환자안전간호활동 결과로 인해 투약오류경험, 환자안전사고 보고 건수가 낮아진 것으로 유추해볼 수 있다. 그 이유로 본 연구에서 임상경력은 5년 이상 집단이 전체 간호사 중 51.7%로 가장 큰 비율을 차지하였는데, 연차가 쌓이면서 독립적인 문제 해결과 위기관리에 숙달된 것이 영향을 미쳤을 것으로 사료된다. 그러나 자율적으로 이루어지는 환자안전사고 설문조사를 통한 자가 보고는 설문지 응답과 실제 수행 간에 오차가 발생할 수 있으므로 반복 연구가 필요하다.

환자안전간호활동에 영향을 미치는 요인에서 가장 영향력 있는 요인은 환자안전역량이며, 설명력이 50.7%로 나타났다. 본 연구대상자의 환자안전역량은 직위와 환자안전관련업무에 통계적으로 유의한 차이를 보이며, 수간호사 그룹과 환자안전관련업무 경험이 없다고 응답한 자에게 환자안전역량이 높은 것으로 조사되었다. 이는 환자안전사고보고와 관련된 업무는 책임간호사 이상에서 다루는 경향이 있어 수간호사는 많은 사건을 간접적으로 경험하며 자연스럽게 환자안전 역량의 강화가 이루어진 것으로 사료된다. 이는 선행연구에서 연령과 총 임상 근무경력이 많고, 의료기관 인증경험이 있는 경우에 간호역량이 향상되고, 직위가 높을수록, 임상경력이 10년 이상, 특수부서에 근무하는 간호사일수록 환자안전관리 중요성을 높게 인식한다는 선행연구의 결과를 뒷받침한다[24]. 또한 상급병원 간호사[25], 간호·간병통합서비스 병동 간호사[22], 중환자실 간호사[26], 수술실 간호사[27]를 대상으로 한 연구결과와도 일치하는 결과이다.

본 연구의 대상자는 환자안전관련 업무경험에 ‘없다’로 응답한 자가 95.2%으로 높은 비율을 보였다. 상급종합병원은 환자안전관련 교육과 지속적인 평가가 이루어지는데 반하여, 미인증 종합병원의 경우 인력과 환자안전간호활동을 위한 규제 및 제도와 구조적 시스템을 불충분하고, 환자안전관련 업무 경험이 낮다. 또한 환자안전 관리 중요성 인식 개선을 위한 교육과 평가 등이 미비한 것이 영향을 주었을 것이라 사료된다. 이는 비록 선행연구 결과와는 다른 결과이지만[22,24-27], 낮은 연차의 간호사가 기술과 지식에 있어서는 비교적 낮은 점수를 보였더라도 환자안전사고 예방에 주의를 기울여 태도에 높은 점수를 보인 것이 환자안전역량 측정 결과에 영향을 미쳤을 것으로 본다. 단, 연구의 범위에 특수부서에 근무하는 간호사는 제외하는 등 제한된 범위의 연구참여자의 특성을 고려하여 성급한 연구결과의 일반화에 주의를 요한다. 현장의 목소리를 따르면, 환자안전역량을 향상시키기 위해 환자안전교육을 제공하는 환자안전전담자들은 환자안전사건 분석 및 개선활동만으로 환자안전교육을 진행하기 쉽지 않다고 하였다[28]. 병원급 이상 의료기관은 자율적으로 의료법 제58조의 4 의료기관 인증의 신청 및 평가에 따라 인증을 신청할 수 있으나 대다수의 종합병원 기관들은 시간과 비용부담으로 인해 자발적 참여가 저조한 실정이다[2]. 그러므로 국가 차원에서 미인증 종합병원 내 환자안전역량을 향상시키기 위해 환자 수에 따른 전담인력 배치 제도 도입이 필요하다. 또한 경력 별로 환자안전사고 발생 시 대처 방법 및 보고 현황을 분석하여 중재안을 전략적으로 개발하는 것이 요구된다.

본 연구를 통해 미인증 종합병원간호사의 환자안전간호활동에 환자안전역량, 환자안전관리 중요성 인식이 영향을 미치는 요소임을 확인하였다. 따라서 향후에는 병원의 규모나 의료기관 인증 상태 등 조직의 특성을 고려한 환자안전간호활동과 관련한 다양한 영향요인을 규명하는 연구가 지속적으로 확대되어야 할 것이다. 또한 미인증 종합병원간호사의 환자안전간호활동 향상을 위하여 영향요인을 반영한 교육프로그램이 운영될 수 있도록 행정적, 재정적 지원이 뒷받침되어야 할 것이다.

## 결론

본 연구는 전라남도와 광주광역시 소재 병원인증평가 미인증 종합병원 5곳에서 환자에게 직접 간호를 제공하고 있는 병동 간호사 230명을 대상으로 종합병원간호사의 환자안전관리 중요성 인식, 환자안전역량이 환자안전간호활동에 미치는 영향을 파악하고자 시도되었다.

본 연구결과를 통하여 환자안전역량, 환자안전관리 중요성 인식, 투약오류경험 유무가 환자안전간호활동에 영향을 미치는 것으로, 설명력은 50.7%로 나타났다. 미인증 종합병원간호사의 환자안전간호활동을 증진시키기 위하여 환자안전역량, 환자안전관리 중요성 인식 향상을 위한 교육 및 인식 개선을 위한 지속적이고 체계적인 관리 전략을 개발하는 것이 요구된다.

향후 개인적 측면으로 환자안전간호활동에 영향을 준 환자안전역량, 환자안전관리 중요성 인식 정도를 높일 수 있도록 연령과 직위, 환자안전관련 업무경험, 환자안전사고 보고 건수와 종류를 고려한 단계적 교육프로그램 중재 개발 및 적용이 필요하다. 의료기관 측면에서는 이를 고려한 병원 내 환자안전간호활동을 돕는 시스템 구축과 환자안전역량을 강화시키는 프로그램의 운영을 촉진시켜야 할 것이다. 국가측면에서는 병원 내에서 환자안전역량 및 중요도 인식 제고를 위한 지속적인 지원체계 구축 및 운영이 가능하도록 재정적, 정책적 지원이 요구된다.

## Figures and Tables

**Table 1.** Participants’ Demographics and Experiences of Patient Safety Issues (N = 230)

Variables	Categories	n	(%)	M ± SD
Sex	Female	221	96.1	
	Male	9	3.9	
Age (yr)	〈 30	99	43.0	32.17 ± 6.08
	30–39	94	40.9	
	≥ 40	37	16.1	
Marital status	Single	155	67.4	
	Married	75	32.6	
Education	College	79	34.3	
	University	151	65.7	
Clinical career (yr)	〈 1	19	8.3	3.45 ± 1.30
	1–2	43	18.7	
	3–4	49	21.3	
	5–9	54	23.5	
	≥ 10	65	28.2	
Position	Nurse	196	85.2	
	Charge nurse	16	7.0	
	Head nurse	18	7.8	
Shift-working pattern	3 shifts	186	80.9	
	Day/evening	13	5.7	
	Full time	31	13.4	
Experience of patient safety education	Yes	205	89.1	
	No	25	10.9	
Number of reported safety incidents over the past 12 months (times)	0	60	26.1	
	1~2	125	54.3	
	3~5	37	16.1	
	6~10	8	3.5	
Fall down experience	Yes	84	36.5	
	No	146	63.5	
Medication error experience	Yes	75	32.6	
	No	155	67.4	
Testing error experience	Yes	20	8.7	
	No	210	91.3	
Surgical procedure errors during treatment	Yes	14	6.1	
	No	216	93.9	
Patient safety-related work experience	Yes	11	4.8	
	No	219	95.2	

M = mean; SD = standard deviation.

**Table 2.** Level of Awareness of the Importance of Safety Management, Patient Safety Competency, and Patient Safety Management Activities (N = 230)

Characteristics	Categories	n	Min	Max	M ± SD
Awareness of the importance of patient safety		21	2.57	5.00	3.92 ± 0.47
	Awareness of patient safety	4	3.00	5.00	4.27 ± 0.49
	Interest in patient safety	7	2.29	5.00	3.85 ± 0.56
	Commitment to patient safety	5	2.40	5.00	3.91 ± 0.53
	Confidence in patient safety	5	2.00	5.00	3.74 ± 0.62
Patient safety competency		41	2.95	5.00	3.92 ± 0.42
	Patient safety attitude	14	3.00	5.00	4.29 ± 0.41
	Patient safety skill	21	2.57	5.00	3.83 ± 0.50
	Patient safety knowledge	6	2.00	5.00	3.35 ± 0.69
Patient safety management activities		32	2.78	5.00	4.30 ± 0.43
	Hand hygiene and infection control	5	3.00	5.00	4.48 ± 0.49
	Accurate patient identification	4	2.75	5.00	4.38 ± 0.51
	Medication	6	2.67	5.00	4.34 ± 0.51
	Fall prevention activities	6	2.33	5.00	4.33 ± 0.52
	Patient safety before surgery/procedure	3	2.67	5.00	4.30 ± 0.62
	Facility and medical equipment management	2	1.50	5.00	4.19 ± 0.69
	Communication	4	2.75	5.00	4.18 ± 0.58
	Fire safety and emergency management	2	1.00	5.00	3.89 ± 0.73

Min = minimum; Max = maximum; M = mean; SD = standard deviation.

**Table 3.** Differences in Awareness of the Importance of Safety Management, Patient Safety Competency, and Patient Safety Management Activities according to General Characteristics (N = 230)

Variables	Categories	Awareness of the importance of safety management		Patient safety competency		Patient safety management activities	
		M ± SD	t/F (*p)*	M ± SD	t/F (*p)*	M ± SD	t/F (*p*)
			Scheffé		Scheffé		Scheffé
Sex	Female	3.91 ± 0.47	-1.20 (.231)	3.82 ± 0.43	-0.79 (.431)	4.31 ± 0.43	-0.60 (.550)
	Male	4.10 ± 0.49		3.93 ± 0.41		4.39 ± 0.34	
Age (yr)	〈 30^a^	3.93 ± 0.46	3.22 (.042)	3.87 ± 0.43	2.22 (.111)	4.37 ± 0.43	2.18 (.116)
	31∼39^b^	3.84 ± 0.46	c > b	3.75 ± 0.40		4.24 ± 0.43	
	≥ 40^c^	4.07 ± 0.50		3.88 ± 0.49		4.31 ± 0.41	
Marital status	Single	3.91 ± 0.50	-0.47 (.639)	3.83 ± 0.45	0.65 (.516)	4.31 ± 0.44	-0.17 (.864)
	Married	3.94 ± 0.41		3.50 ± 0.39		4.32 ± 0.40	
Education	College	3.90 ± 0.45	-0.35 (.727)	3.79 ± 0.44	-0.72 (.471)	4.30 ± 0.46	-0.15 (.882)
	University	3.92 ± 0.48		3.84 ± 0.43		4.31 ± 0.41	
Clinical career (yr)	〈 1	3.98 ± 0.52	1.90 (.111)	3.81 ± 0.56	1.10 (.357)	4.25 ± 0.49	1.25 (.292)
	1∼2	4.00 ± 0.53		3.89 ± 0.48		4.35 ± 0.48	
	3∼4	3.76 ± 0.40		3.72 ± 0.31		4.21 ± 0.37	
	5∼9	3.94 ± 0.45		3.86 ± 0.41		4.39 ± 0.41	
	≥ 10	3.95 ± 0.47		3.83 ± 0.45		4.31 ± 0.42	
Position	Nurse^a^	3.90 ± 0.46	4.11 (.018)	3.80 ± 0.43	3.64 (.028)	4.31 ± 0.43	1.07 (.343)
	Charge nurse^b^	3.79 ± 0.45	c > b	3.86 ± 0.40	c > a	4.20 ± 0.45	
	Head nurse^c^	4.20 ± 0.48		4.08 ± 0.44		4.42 ± 0.38	
Shift-working pattern	3 shifts	3.90 ± 0.46	0.85 (.430)	3.82 ± 0.43	0.21 (.810)	4.31 ± 0.44	0.05 (.953)
	Day/evening	3.94 ± 0.39		3.89 ± 0.29		4.28 ± 0.35	
	Full time	4.02 ± 0.54		3.80 ± 0.47		4.30 ± 0.42	
Education experience of	Yes	3.93 ± 0.46	0.84 (.404)	3.83 ± 0.43	1.03 (.306)	4.32 ± 0.41	1.29 (.197)
patient safety	No	3.84 ± 0.54		3.74 ± 0.43		4.20 ± 0.53	
Number of reported safety incidents over the past 12 months (times)	0^a^	3.97 ± 0.48	0.83 (.479)	3.86 ± 0.49	0.68 (.563)	4.42 ± 0.42	3.85 (.010)
	1~2^b^	3.92 ± 0.47		3.81 ± 0.42		4.30 ± 0.42	d > c
	3~5^c^	3.82 ± 0.48		3.77 ± 0.40		4.13 ± 0.45	
	6~10^d^	3.94 ± 0.45		3.97 ± 0.34		4.40 ± 0.32	
Fall down experience	Yes	3.87 ± 0.46	1.85 (.065)	3.79 ± 0.43	1.40 (.161)	4.31 ± 0.43	-0.26 (.796)
	No	3.99 ± 0.47		3.87 ± 0.43		4.30 ± 0.43	
Medication error experience	Yes	3.87 ± 0.54	1.00 (.321)	3.77 ± 0.43	1.20 (.233)	4.17 ± 0.45	3.50 (.001)
	No	3.94 ± 0.43		3.85 ± 0.43		4.38 ± 0.41	
Testing error experience	Yes	3.87 ± 0.49	0.42 (.675)	3.82 ± 0.34	0.02 (.986)	4.17 ± 0.35	1.51 (.132)
	No	3.92 ± 0.47		3.82 ± 0.44		4.32 ± 0.43	
Surgical procedure errors during treatment	Yes	3.98 ± 0.46	-0.52 (.604)	3.73 ± 0.44	0.87 (.387)	4.31 ± 0.37	-0.04 (.971)
	No	3.91 ± 0.47		3.83 ± 0.43		4.31 ± 0.43	
Patient safety-related work experience	Yes	3.91 ± 0.47	0.36 (.723)	3.81 ± 0.42	2.57 (.011)	4.30 ± 0.43	1.05 (.297)
	No	3.97 ± 0.56		4.14 ± 0.45		4.44 ± 0.43	

M = mean; SD = standard deviation.

**Table 4.** Correlations among Awareness of the Importance of Safety Management, Patient Safety Competency, and Patient Safety Management Activities (N = 230)

Variables	Awareness of the importance of safety management	Patient safety competency	Patient safety management activities
	r (*p*)		
Patient safety competency	.62 (< .001)	1	-
Patient safety management activities	.60 (< .001)	.64 (< .001)	1

**Table 5.** Factors Affecting Participants’ Patient Safety Management Activities

Variables	Model Ⅰ							Model Ⅱ						
	B	SE	β	t	*p*	95% CI	VIF	B	SE	β	t	*p*	95% CI	VIF
(Constant)	4.44	0.05		81.85	< .001			1.63	0.20		8.26	< .001		
Reported incidents for the past 12 months (ref = 0)	-0.10	0.07	-0.10	-1.54	.125	1.87-2.07	1.09	-0.07	0.05	-0.07	-1.49	.137	1.87-2.07	1.09
Medication error experience (ref = yes)	-1.79	0.06	-0.20	-2.92	.004	0.26-0.39	1.09	-0.14	0.04	-0.15	-3.09	.002	0.26-0.39	1.09
Awareness of the importance of patient safety management								0.28	0.05	0.31	5.21	< .001	3.85-3.98	1.66
Patient safety competency								0.44	0.06	0.44	7.32	< .001	3.77-3.88	1.66
F (*p*)	7.34 (< .001)							59.83 (< .001)						
R^2^	6.1%							51.5%						
Adjusted R^2^	5.2%							50.7%						
Durbin-Watson	1.9							1.9						

CI = confidence interval; VIF = variance inflation factor; ref = Reference.

## Data Availability

Please contact the corresponding author for data availability.
